# Human Oral Mucosa Stem Cells Increase Survival of Neurons Affected by In Vitro Anoxia and Improve Recovery of Mice Affected by Stroke Through Time-limited Secretion of miR-514A-3p

**DOI:** 10.1007/s10571-022-01276-7

**Published:** 2022-09-09

**Authors:** Paula Stančin, Min Suk Song, Ivan Alajbeg, Dinko Mitrečić

**Affiliations:** 1 Laboratory for Stem Cells, Croatian Institute for Brain Research, University of Zagreb School of Medicine, Zagreb, Croatia; 2 Omnion Research International, Zagreb, Croatia; 3 Department of Oral Medicine, University of Zagreb School of Dental Medicine and University Hospital Centre Zagreb, Zagreb, Croatia

**Keywords:** Human oral mucosa stem cells, Anoxia, Stroke, miR-514A-3p

## Abstract

The success rate of regenerative medicine largely depends on the type of stem cells applied in such procedures. Consequently, to achieve the needed level for clinical standardization, we need to investigate the viability of accessible sources with sufficient quantity of cells. Since the oral region partly originates from the neural crest, which naturally develops in niche with decreased levels of oxygen, the main goal of this work was to test if human oral mucosa stem cells (hOMSC) might be used to treat neurons damaged by anoxia. Here we show that hOMSC are more resistant to anoxia than human induced pluripotent stem cells and that they secrete BDNF, GDNF, VEGF and NGF. When hOMSC were added to human neurons damaged by anoxia, they significantly improved their survival. This regenerative capability was at least partly achieved through miR-514A-3p and SHP-2 and it decreased in hOMSC exposed to neural cells for 14 or 28 days. In addition, the beneficial effect of hOMSC were also confirmed in mice affected by stroke. Hence, in this work we have confirmed that hOMSC, in a time-limited manner, improve the survival of anoxia-damaged neurons and significantly contribute to the recovery of experimental animals following stroke.

**Supplementary Information:**

The online version contains supplementary material available at 10.1007/s10571-022-01276-7.

## Introduction

Oral cavity is a rich source of stem cells that can be collected with a relatively simple procedure. Thus far, stem cells isolated from dental pulp of permanent and deciduous teeth, dental follicle, dental bud, apical papilla, periodontal ligament and oral mucosa have been described (Egusa et al. 2012). Oral mucosa is a rather newly described source of human oral mucosa stem cells (hOMSC), with its *lamina propria* harbouring a cell population which expresses embryonic stem cell transcription factors Oct4, Sox2 and Nanog. Furthermore, these cells also exhibit potential for differentiation into ectoderm, mesoderm and endoderm (Marynka-Kalmani et al. 2010; Dupin and Coelho-Aguiar 2013; Miyoshi et al. 2015; Alajbeg et al. 2018).

Since the hOMSC population expresses markers of pluripotent embryonic stem cells as well as multipotent mesenchymal stem cell markers, their therapeutic potential has been demonstrated in several studies. In rats with sciatic nerve injury, transplantation of hOMSC yielded improved motor function (Ganz et al. 2014a, b), while in the Parkinson's disease model, they improved the symptoms of the disease (Ganz et al. 2014a, b). A similar finding was described in a rat model of spinal cord injury-induced neurogenic bladder (Cho et al. 2014). Equally, hOMSCs have been successfully differentiated into osteoprogenitors and applied to close calvaria defects (Lopez-Letayf et al. 2022). Mouse OMSCs were successfully used in treating of wounds in a diabetic mouse model (Kuperman et al. 2020). Although molecular basis of all these observed therapeutic phenomena remains elusive, some articles suggested that at least a part of their beneficial effects was achieved over secretion of GFAP, VEGF and BDNF (Ganz et al. 2014a, b). On the other hand, in cases when hOMSC differentiate into specific neuronal subpopulation, like for example in dopaminergic neurons, their beneficial effects in Parkinsonian model was assigned to production of dopamine (Ganz et al. 2014a, b).

Ischemic injury of the nervous tissue is the main cause of death and lifelong disability in the Western world (Radak et al. 2017). Although there has been some progress in the prevention and treatment of stroke, therapeutic options remain limited. One of the new approaches which brought hope is the application of therapeutic potential of stem cells. Indeed, recent preclinical and clinical studies reported measurable levels of success (Wang et al. 2018; Zhang et al. 2022). The main goal of this work was to test hypothesized protective effects of hOMSC on ischemic injury of the neural cells. For this purpose, we utilized co-cultures of human neuronal cells obtained from iPSC and hOMSCs. Moreover, hOMSC have been transplanted to the MCAO mouse model of stroke followed by assessment of behavioural improvements. In addition, here we show that at least a part of discovered beneficial effects of hOMSC on neurons affected by anoxia are achieved over miR-514A-3p, miRNA which has, thus far, not been considered much with respect to the nervous tissue. Results of this study open a possibility that hOMSC, which are a rather easily accessible source of human stem cells, might represent a very potent candidate for clinical trials on patients affected by stroke.

## Materials and Methods

### Oral Mucosa Biopsy

Based on the approval obtained from the Ethical Board of the University of Zagreb School of Medicine, four volunteers, aged between 22 and 24 years old, signed a statement of informed consent. The biopsy procedure started with injection of 2% lidocaine anaesthesia (Lidocaine 2%, JGL). 10 min following the anaesthesia, the mucosa was immobilized with tweezers. This was followed by a punch biopsy through which a piece of tissue with a volume of 3 × 3 × 3 mm was obtained. The biopsy site was closed with a resorptive suture. The procedure was approved by the Ethical Board of the School of Medicine (approval No. 380-59-10106–17-100/27).

### Isolation and Cultivation of hOMSC

Obtained oral mucosa tissue was briefly washed in a medium with added streptomycin, penicillin and amphotericin B (LG-DMEM, 12320032; P&S, 15140122; Amphotericin B, 15290018, everything Gibco) cut into small pieces and then immersed in the mixture of collagenase and dispase (Merck, 10269638001, 5 mg/ml) overnight. The following day, lamina propria was mechanically separated from the epithelium, cut into pieces of approx. 0.5 mm and plated in six-well plates with growth medium comprising LG-DMEM, 10% FBS, 1% Glutamax and antibiotics. The plates were kept at 37 °C and 5% CO_2_ in a humidified culture incubator. The growth medium was partially changed every third day. 15 days following the plating, cells growing from the pieces of tissue have been noticed and were allowed to cover 60–70% of the plate surface. When cells reached the desired confluence, they were detached using 0.25% trypsin and seeded in new plates. For these experiments, cells between the 2nd and the 7th passage were used. For all the experiments described in this article, cells originating from the same donor were used.

### Cultivation of hIPSC, Obtaining of Neurons and Co-cultivation Experiments

A commercially obtained human IPSC line WTSIi189-A was used in experiments pertaining to anoxia and for obtaining neurons. Neurons were differentiated through the following procedure: after plating the cells on Matrigel-coated well plates in E8 medium, supplemented with ROCK inhibitor analogue (Stem Cell Technologies, Y-27632), neural induction was initiated by addition of N2B27 medium (1:1 mixture of N2- and B27-containing media). The N2 medium comprised DMEM/F12 (Gibco, 11330032), N2 (Gibco, 17502048), 5 μg/ml insulin (Sigma, I9278), glutamax, non-essential amino acids, 2-mercaptoethanol (Sigma, 8057400005), penicillin/streptomycin. On the other hand, the B27 medium consisted of Neurobasal (Gibco, 21103049, B27 without the retinoic acid (12587010), 1 × glutamax, 1 × 200 mM glutamine and 1 × penicillin/streptomycin, supplemented with SB431542 10 μm (Tocris) and LDN193189 1 μM (Miltenyi Biotec). After dissociation with Dispase, cells were replated in the N2B27 medium with 20 ng/μl bFGF (Peprotech). For terminal neuronal differentiation, NPCs were replated on DIV35 on poly-ornithine and laminin-coated plastic dishes in N2B27 medium. DIV45-50 was used for these experiments. For all experiments in which co-cultures were needed, transwell systems with a pore diameter of 3.0 µm were used. Co-cultures of hOMSC and hIPSC were grown for 28 days, with measurements being obtained on Days 0 (a day before establishing the co-cultures), Day 14 and Day 28.

### Immunostaining and Analyses of Signal Intensity

For the purpose of performing immunocytochemical staining, the cells were seeded on glass slides placed within 24 well plates, fixed with 4% PFA for 15 min and then washed 3 × 5 min with PBS. Cells were permeabilized with a 0.2% Triton detergent (Triton, Sigma; 44 μl in 22 ml PBS) for 10 min and blocked in 3% goat serum (Sigma) for 2 h. For the purpose of obtaining histological sections of mouse brain, after perfusion with 4% PFA, tissue was sectioned on cryomicrotome on 20 µm thick sections. Primary antibodies were used against the following epitopes: NES (ab6320), 1:200; OCT4 (ab181557), 1:250; Map2 (Mab3418), 1:400. The incubation was performed at 4 °C overnight. Secondary antibodies used included Alexa Fluor 488 (LifeTechologies, A11001) and Alexa Fluor 546 (LifeTechologies, A11040), 1:1000 which were incubated for a total of 2 h. The slides were stained for 10 min with DAPI (1:8000) and then covered with Dako Fluorescent Mounting Medium (S3023).

For performing the analyses of signal intensity the CellProfiler software was used. For this purpose, we used three different brains and scanned 15 images for each brain focusing on the region of penumbra. After manually preparing a pipeline with an acceptable signal–noise ratio, all the images were analysed with an automatic protocol as a single batch. After obtaining the raw data for percentage of slide coverage and mean intensity as well as colocalization through image analysis the parameters were statistically analysed using a two-way ANOVA with Tukey Kramer multiple comparisons post-hoc test.

### qPCR

Total RNA was extracted from cell cultures by RNeasy Mini Kit (Qiagen). The RNA quality was assessed using 2100 Bioanalyzer (Agilent) and NanoDrop spectrophotometer (Thermo Scientific). qRT-PCR analyses was performed using 7500HT instrument (Applied Biosystems). Total RNA was reverse transcribed with specific primers and then qRT-PCR was performed with the following TaqMan Assays (Thermo Fisher Scientific): Mm02619580_g1 for Actb, Hs04260367_gH for OCT4, Hs04234836_s1 for SOX2, Hs01002915_g1 for CD40, Hs00977641_m1 for CD166, Hs06633377_s1 for CD90, Hs00159686_m1 for CD73, Hs04187831_g1 for NES, Hs00161904_m1 for SNAI2, Hs02718934_s1 for BDNF, Hs00900055_m1 for VEGF, Hs00171458_m1 for NGF, Hs01931883_s1 for GDNF and Hs04940643_m1 for miR-514a-3p. All target genes were assayed in triplicates on each plate and all qPCR experiments were independently repeated five times.

### Analyses of Cell Viability

Cell viability and damage was quantified by counting of the total number of cells, by Live Dead kit and by an LDH assay. The counting of cells was performed using a Cell Counting kit-8 (Sigma, 96992), cell death was visualized by Live Dead (Thermofisher L3224) and the LDH assay was performed using a LDH kit (Abcam, ab65393). All quantifications were performed by analysing five visual fields in three independent experiments.

### Overexpression and Inhibition of miR-514-3p and Inhibition of SHP-2

To achieve an increased expression of miR-514a-3p, the cells were transfected with 10 pmol/ml of miR mimics (MISSION microRNA mimics, Sigma Aldrich, HMI0652) using Lipofectamine LTX (Life Technologies). A non-specific miRNA sequence served as a control. For miR-514a-3p inhibition experiments, the cells were transfected with 10 pmol/ml of inhibiting miRNA sequence (MISSION microRNA inhibitors, Sigma Aldrich, HLTUD0650) using Lipofectamine LTX transfection reagent (Life Technologies). A non-specific miRNA sequence (MISSION microRNA inhibitor; Sigma Aldrich) served as a control for inhibiting sequence. To block SHP-2, 10 mM of PHPS1 was added to the cell culture (Bio-Techne, 6771).

### Induction of Stroke and Transplantation of hOMSCs

All the experiments were performed in accordance with the obtained ethical approval while strictly adhering to the regulatory guidelines. Mice (strain C57Bl/6 N) were kept at a temperature of 22 °C, relative humidity of 50% ± 10% and with a light/dark cycle of 12/12 h. Pelted food and water were given ad libitum. The induction of ischemic brain stroke was performed under inhalation anaesthesia using 2% isoflurane-piramal. After a midline incision was made on the ventral neck region, a filament was inserted through the common carotid artery (CCA), into the internal carotid artery (ICA) and trough circle of Willis to occlude the middle cerebral artery (MCA). The occlusion lasted for 45 min, and was followed by filament retrieval and reperfusion. After this microsurgery, the animals received the buprenorphine analgetic (0.003 mg/kg) as well as an intraperitoneal infusion of 0.5 mL of 20% glucose solution in saline and were kept on a heating pad for the next 24 h. The animals’ health condition was assessed by scoring of the following parameters: motility, gait disturbances and reflexes. Only those animals which developed clear signs of stroke (more than 10 points out of maximal 18 for impairment) were used for the transplantation experiments. After starting with 20 animals, only those 10 animals with the most obvious signs of neurological deficits were selected for transplantation experiments. The transplantation was performed 48 h after the induction of MCAO. One group of animals (*n* = 5) received one million of hOMSCs in 1 μl of LG-DMEM medium, while the control group (*n* = 5) received 1 μl of cell medium without cells. Stereotaxic coordinates for transplantation were as follows: AP + 0.5, ML + 1.5, DV − 2.5. For transplantation, an opening in the bone and dura was made by sterilized drill with diameter of 0, 8 mm. For injection of cells, 10 µl Hamilton syringe (Hamilton Company, 7635-01) with Hamilton needle (30 gauge, 7803-07) was used. Injection was performed in the period of 15 min, after which a syringe remained at the place for additional 5 min.

### Assessment of Mice Health Conditions

Three tests for assessment of health condition of mice were used. They included Rotarod, Open field test and Y-maze. The total number of animals used for these tests was 10 (five with transplanted cells and five with transplanted medium). The Rotarod test was used to measure the general health condition of the mice, which involves strength and a proper use of muscles as well as the ability to maintain balance. It was performed using a treadmill with a 3 cm diameter (Muromachi) which was rotated with accelerating velocity (4–40 rpm) for 5 min. The number of seconds the mice were able to maintain their position on the rod without falling was counted. Testing time points were 1, 3, 7, 14 and 30 days after the induction of stroke. The Open field test was used to evaluate the general locomotor activity and the level of fear in the animals. Each mouse was placed at the centre of a box with dimensions of 75 cm × 75 cm (Harvard Apparatus). The floor of the box was divided by 1 cm wide black lines into 25 equal squares. The locomotor activity was defined as maintaining at least three paws in a certain quadrant throughout a 5 min period. Testing time points were: 8, 15 and 30 days after induction of stroke. The Y-maze test was conducted to test the memory and curiosity of the animals. It was performed using a Y-maze which comprised three plastic arms (two of 30 cm and one of 40 cm length). Once the mouse is placed in one arm, it visits another arm. If the mouse then moves from the second arm to the third arm, i.e. if it does not return to the previous arm, this is scored as a spontaneous alteration. The testing was performed in 8 min periods and testing time points were 8, 15 and 30 days after the induction of stroke.

### Statistics

**Fig. 1:**
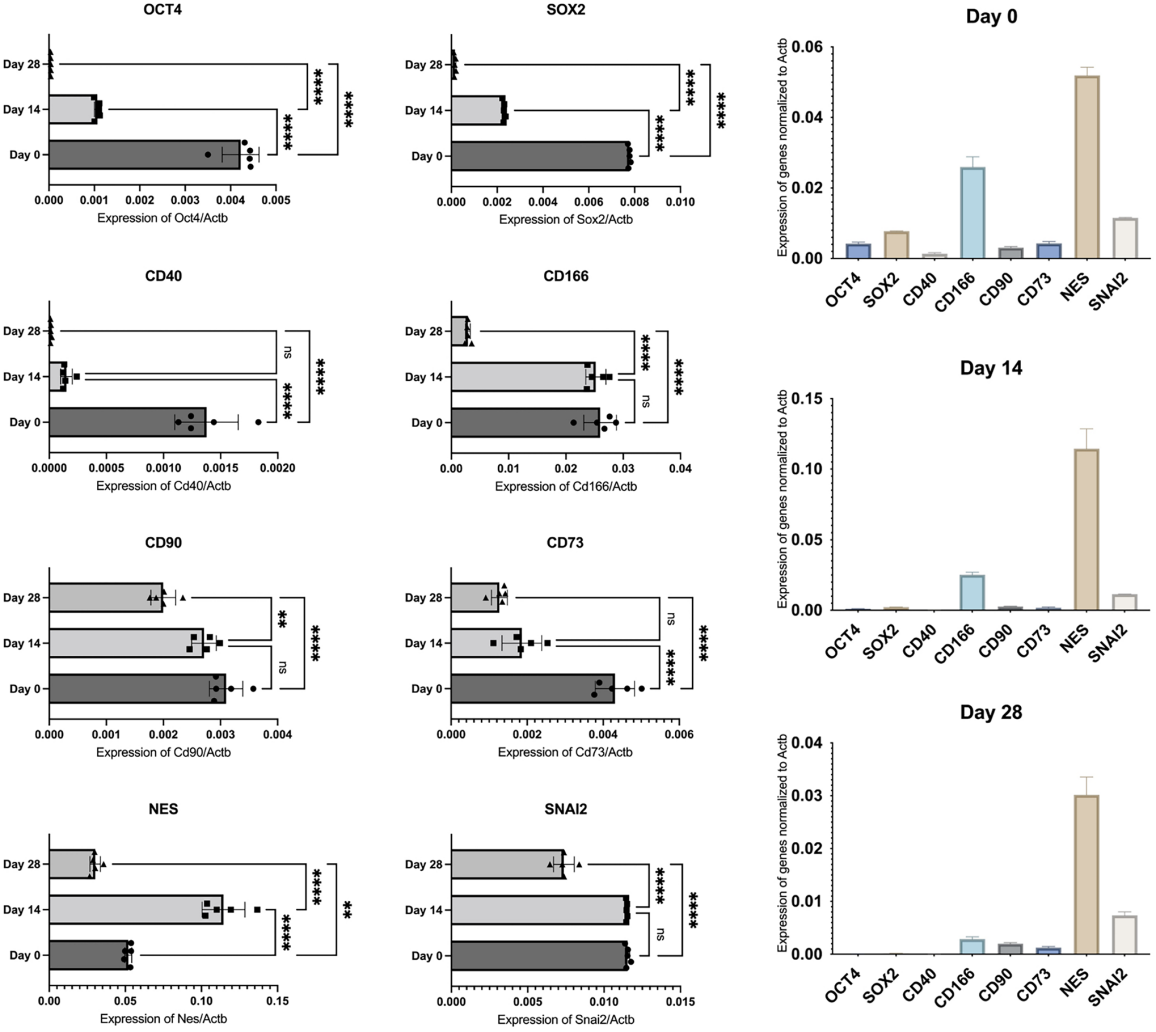
qPCR-assessed expression of genes in 3 time points: a day before establishing the co-culture with differentiated human IPSC-derived neurons and in time points of 14 and 28 days of co-cultivation. As is visible from the analysis, the markers of pluripotency OCT4 and SOX2alongside markers of mesenchymal cells CD166, CD90 and CD73, were clearly positive in the first time point. Positive markers of neural crest cells, SNAI2 and neural progenitors, NES, can also be noticed. On the other hand, the majority of measured markers were downregulated when hOMSCs were co-cultivated with neurons. This is clearly visible on Days 14 and 28

**Fig. 2:**
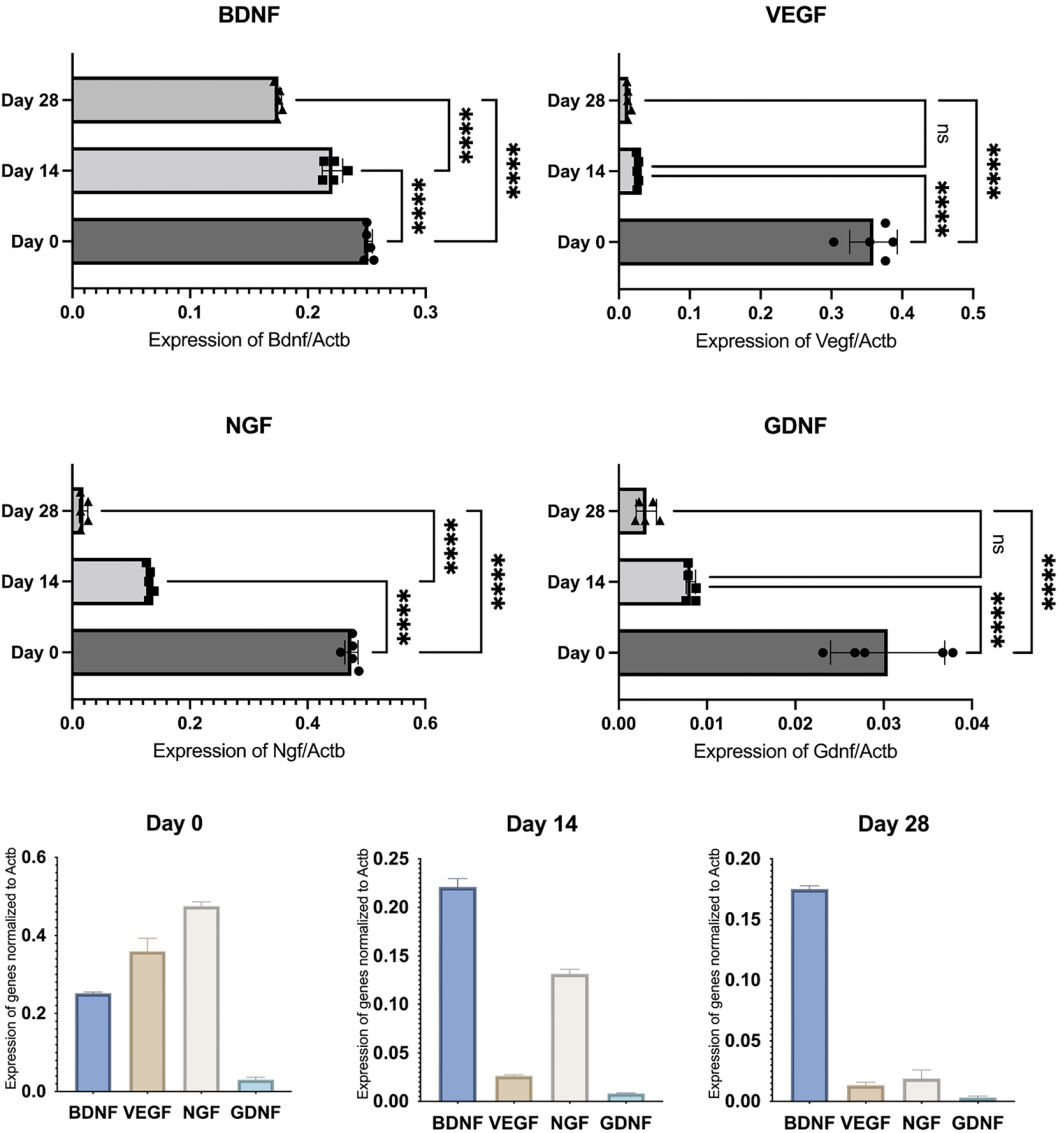
hOMSCs exhibit high expression of BDNF, VEGF and NGF, while GDNF was not so prominent. Expression of all measured growth factors was downregulated when hOMSCs were co-cultivated with human neurons, which was visible on Days 14 and 28

**Fig. 3:**
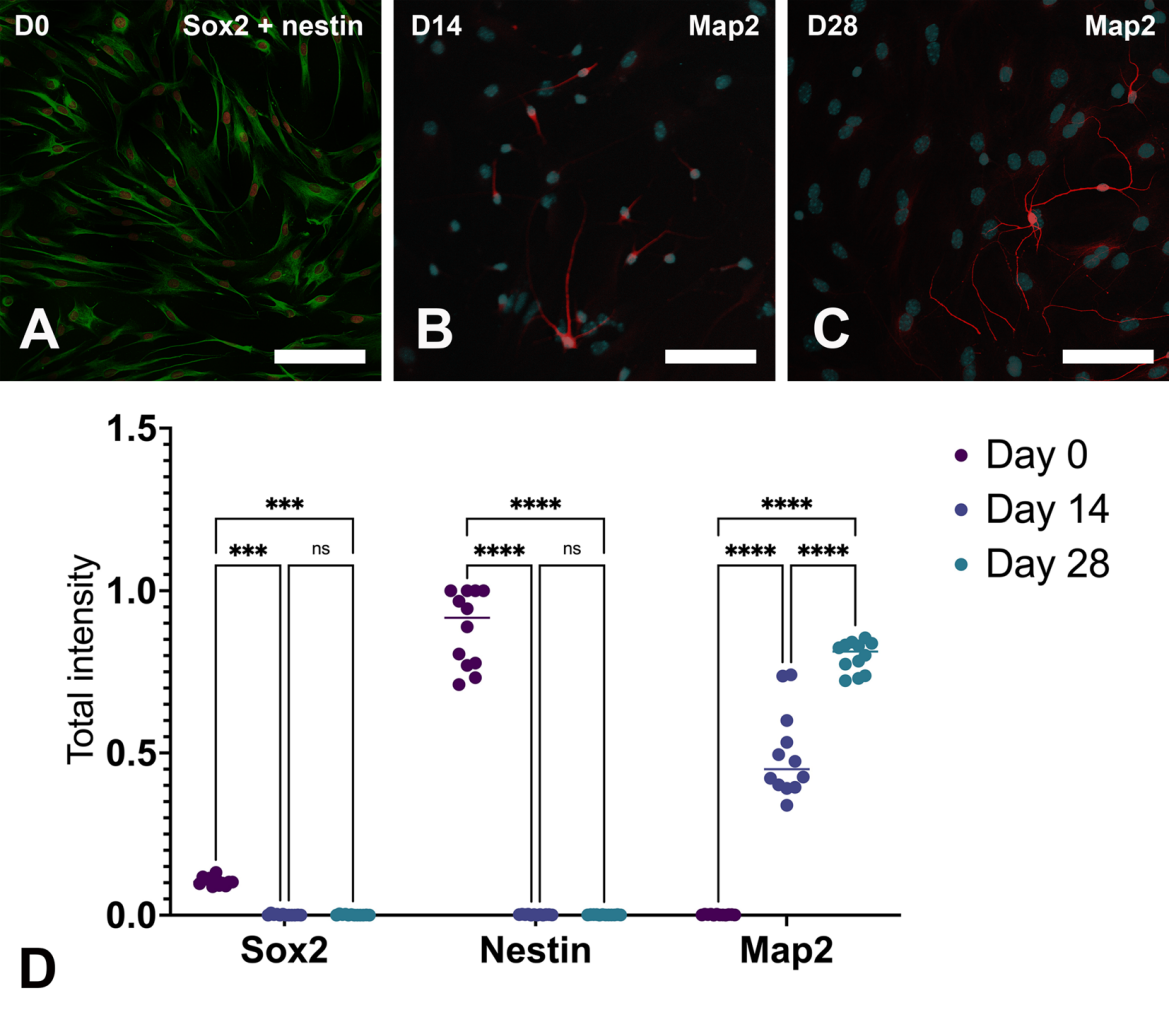
Immunocytochemical analyses revealed that, before being co-cultivated with human neurons, hOMSCs strongly express nestin (green) and SOX2 (red) (3A). After 14 days of co-cultivation with human neurons, some hOMSCs started to make projections, which were Map2-positive (red) (3B). After 28 days of co-cultivation, projections in some cells became even longer (3C). Scale bar: 100 µm

**Fig. 4:**
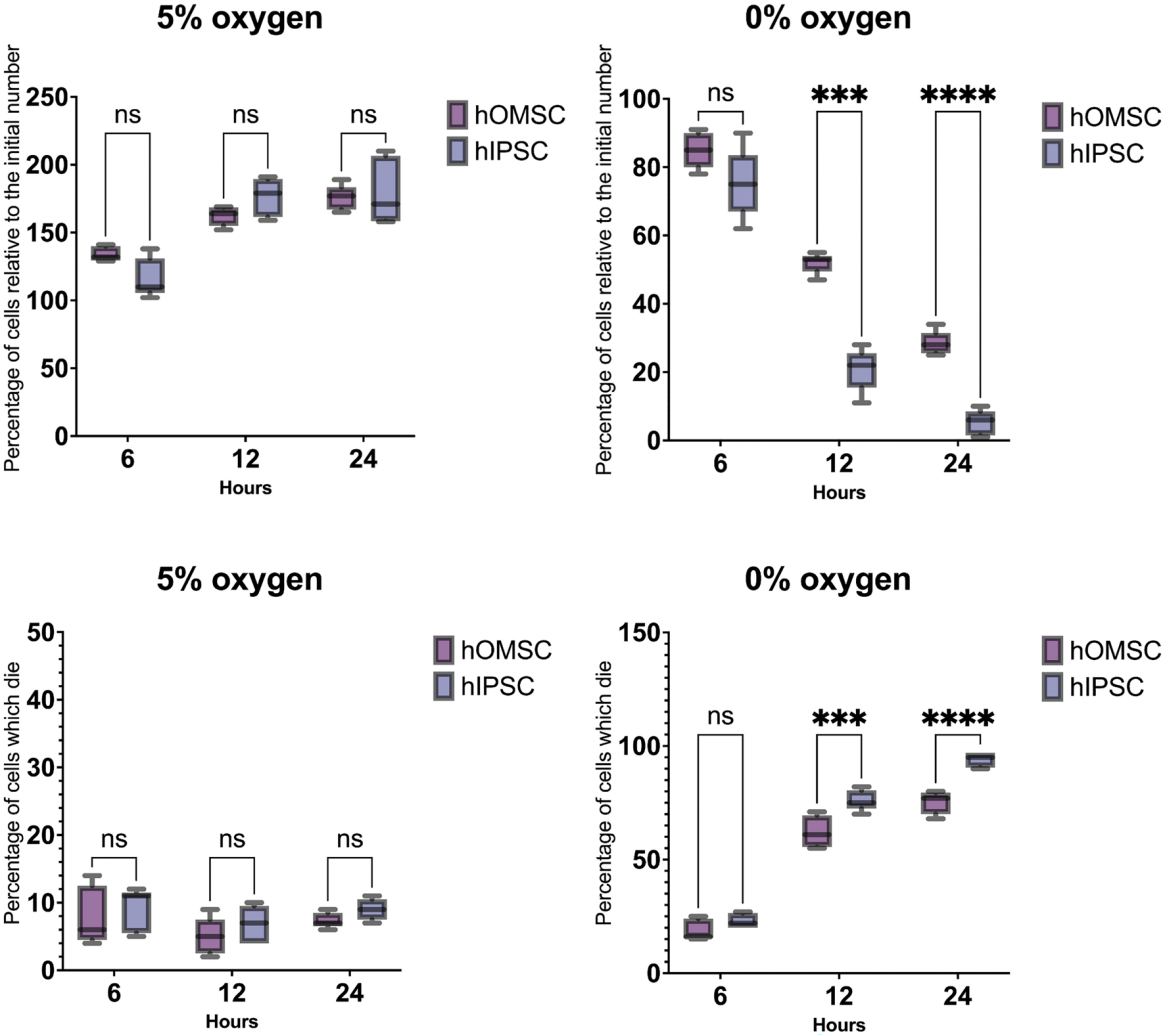
To evaluate the resistance of hOMSCs to anoxia, their survival was compared to hIPSCs in 5% and 0% of oxygen. Both number of cells was measured (by Cell counting kit) and number of dying cells (by Live Dead assay). Even though both cell types benefited from 5% oxygen, there were no significant differences between them. On the other hand, exposure to anoxia revealed that hOMSCs were more resistant than hIPSCs. While no difference in cell survival was observed after 6 h, significantly larger numbers of hOMSCs with less cell death were found 12 and 24 h after the onset of anoxia

**Fig. 5:**
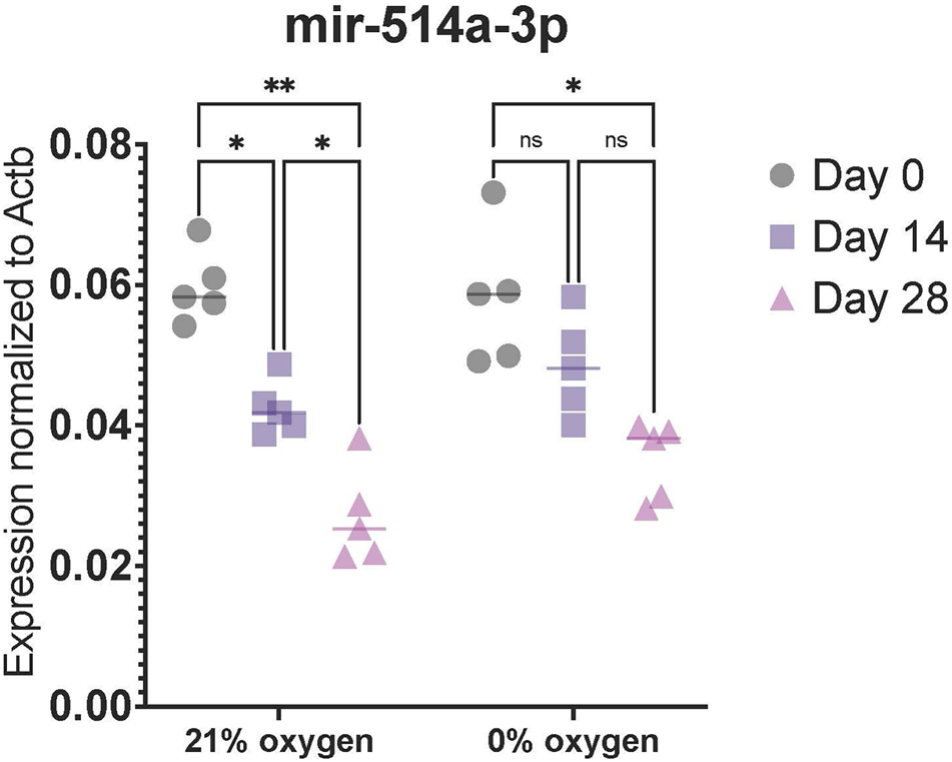
Expression of miR-514a-3p in regard to levels of oxygen and length of cultivation of hOMSCs. It is clearly visible that miR-514a-3p which is downregulated upon 14 and 28 days of contact with human neurons. Both transcription and downregulation are equally present in normoxic and anoxic conditions

Differences in hOMSC gene expression profiles (Figs. 1, 2) as well as cell survival (Fig. 3) and LDH release (Fig. 4) following anoxia and reoxygenation were statistically analysed using a one-way ANOVA with a Tukey Kramer multiple comparisons *post-hoc* test to characterize specific differences between the groups. Statistical analysis pertaining miRNAs expression (Fig. 5) was done using a two-way ANOVA with a Tukey–Kramer multiple comparisons *post-hoc* test.

**Fig. 6:**
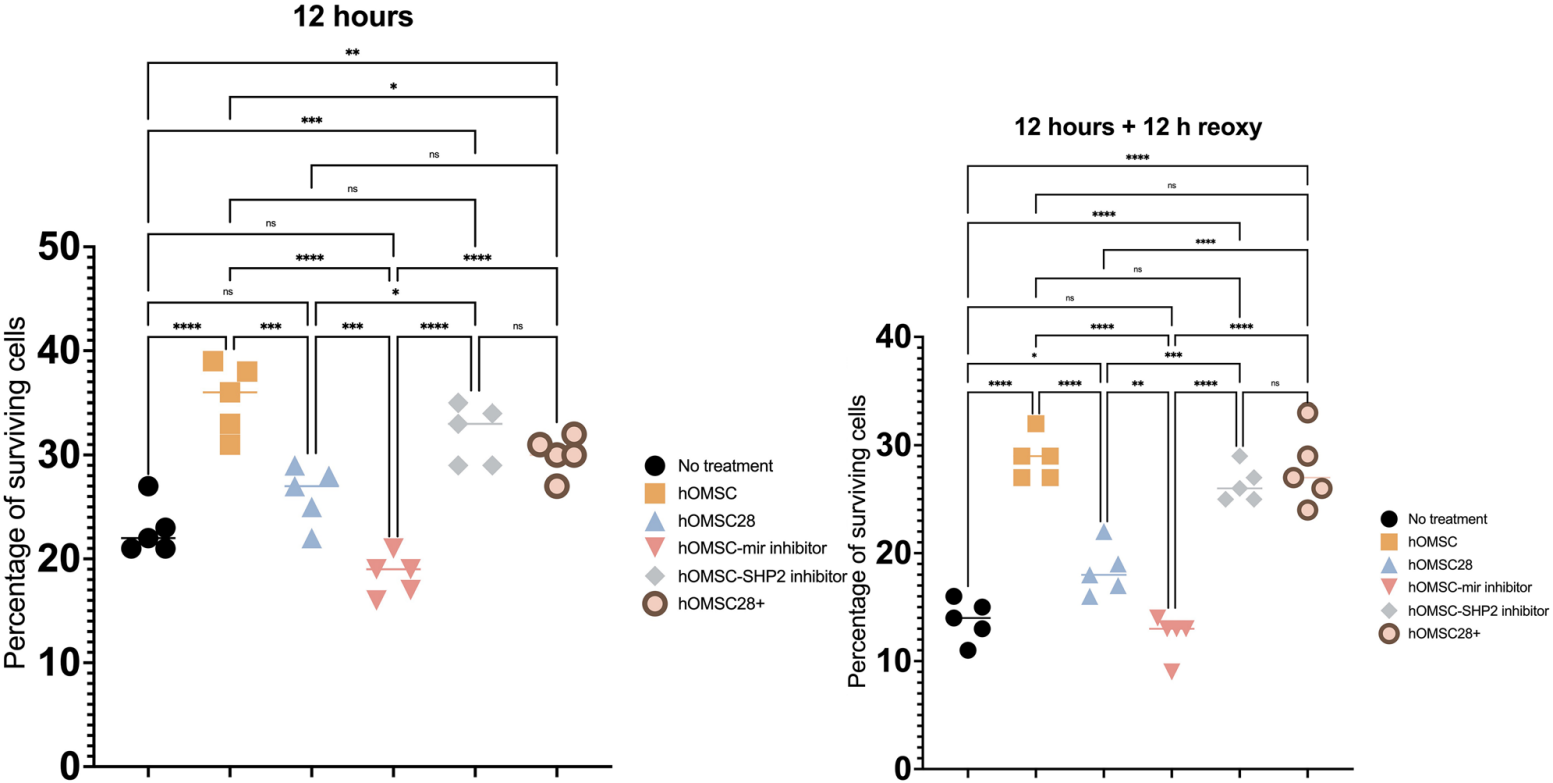
Following the 6 h exposure to anoxia, neurons were placed in contact with hOMSCs and their survival was quantified after 6 h of co-culture. In the group in which no hOMSCs were added (no treatment), the average cell survival was 22%. However, neurons which received help from hOMSCs exhibited significantly improved survival reaching average of 36% (hOMSC). On the other hand, when hOMSCs which were co-cultured with neurons for 28 days were added to neurons, the rescuing effect was not achieved (hOMSC28). The same lack of rescuing effect was found in hOMSCs in which miR-514-3p was inhibited (hOMSC-miR inhibitor). Nevertheless, the rescuing effect was regained when hOMSCs without miR-514-3p were combined with a pharmacological block of SHP-2 (hOMSC-SHP-2), as well as when hOMSCs which have spent in 28 days of co-culture with neurons were modified to start to produce large quantities of miR-514-3p (hOMSC28 +). Very similar effects were detected after 12 h of reoxygenation

**Fig. 7:**
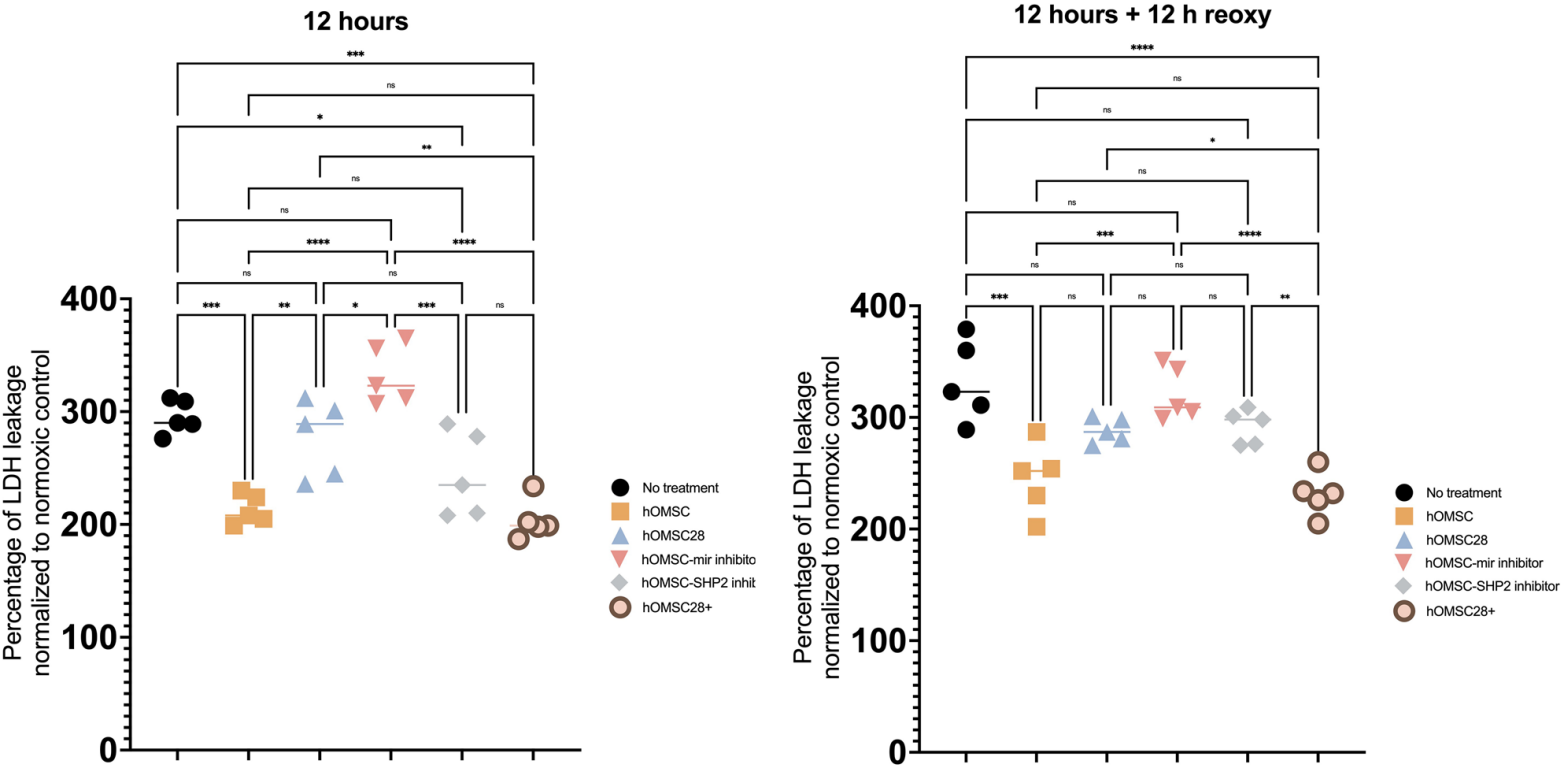
To estimate LDH release, a marker of cell damage, we performed an identical experiment to the one visible in Fig. 6. Following 6 h of anoxia and then 6 h of co-culture with various types of hOMSCs, the levels of released LDH were measured. Again, the addition of hOMSCs significantly benefited the anoxia-damaged cells, reducing LDH release for almost 30%. The rescuing effect was not present when hOMSCs co-cultured with neurons for 28 days (hOMSC28) and hOMSCs without miR-514-3p (hOMSC-miR) were used. The regain of rescuing capacity was only partly returned when a pharmacological block of SHP-2 was used (hOMSC-SHP-2), while hOMSCs co-cultivated with neurons for 28 days in which overexpression of miR-514-3p was induced (hOMSC28 +) reached equally strong effects like the starting group of hOMSCs

The differences in hOMSC and hIPSC survival following exposure to anoxia (Fig. 6) was statistically analysed using a two-way ANOVA with a Geisser–Greenhouse's correction and Šidák's multiple comparisons test. Results obtained by behavioural tests following hOMSC transplantation (Fig. 7) were analysed using two-way ANOVA with Šidák's multiple comparisons test.

The significance levels are as follows: *p < 0.05, **p < 0.01, ***p < 0.001, ****p < 0.0001. Statistical analysis was conducted using GraphPad Prism version 9.3.1 (GraphPad Software, La Jolla, CA, USA).

## Results

### Gene Expression Profile of hOMSCs Depends on the Length of Their Differentiation Promoted by Mature Neurons

Since our first goal was to analyse how hOMSCs faced with neural tissue change their profile over time, we allowed them to mature in the co-culture with human IPSC-derived neurons (DIV50) for 28 days. The expression of following markers was measured by qPCR from RNA originated from isolated hOMSC on Day 0 (a day before the start of the co-culture), Day 14 and Day 28: OCT4, SOX2, CD40, CD166, CD90, CD73, NES and SNAI2. In addition, the expression of four growth factors, including BDNF, VEGF, NGF and GDNF, was measured. All results are shown in Figs. 1, 2.

As expected, cells were, just prior to their placement under the influence of neural tissue, expressing high levels of markers of pluripotency, OCT4 and SOX2, alongside with high levels of markers of their mesenchymal definition, CD166, CD90 and CD73. Concurrently, NES and SNAI2, the markers of neural potential and neural crest origin were clearly positive as well. On the other hand, the CD40 marker of fibroblasts was barely detectable. Simultaneously, three markers important for hOMSCs regenerative potential, including BDNF, VEGF and NGF, were strongly expressed, while GDNF was expressed at much lower levels.

After 14 days of co-cultivation with human neural tissue, another measurement of the same markers was performed. While stressing a strong downregulation of OCT4, SOX2 and CD73, alongside with a strong upregulation of NES, other values are visible in Fig. 1. After 28 days of co-cultivation, many markers decreased to levels of being barely detectable, including the expression of growth factors.

Interestingly, morphological analyses revealed huge differences between the observed three time points (Fig. 3). Prior to exposure to differentiated human neurons, hOMSCs strongly expressed nestin and Sox2 (Fig. 3). This was also visible on the level of RNA expression. After 14 days of co-culture, the total number of hOMSCs was approx. 50% of the starting number, with some cells starting to form projections which were clearly Map2-positive (Fig. 3). After 28 days of co-culture, the number of hOMSCs was the same as on Day 14, but we were able to find some cells with even longer Map2-positive projections.

### hOMSCs Exhibit Higher Resistance to Anoxia Than hIPSCs

Since one of the major goals of this work was to check the potential of hOMSCs for treating of neurological diseases characterized by anoxic damage, we cultivated them in 5% and 0% of oxygen and compared their survival to hIPSCs. As expected, both cell populations benefited from cultivation in 5% of oxygen, so their increased their numbers by 150–200% (Fig. 4). However, when hOMSCs and hIPSCs were exposed to anoxia, hOMSCs appeared much more robust. While 6 h of anoxia slightly influenced both cell populations, a big difference was found after 12 h where around 50% of hOMSCs remained alive, compared to less than 20% of hIPSCs. After 24 h, as expected, the level of survival of hIPSCs was near 0%, while 20% of hOMSCs were still recognized as alive (Fig. 4).

### hOMSCs Express mir-514A-3p and Its Levels Depend on the Length of the Exposure to Mature Neurons

During the screening, in which it was revealed that hOMSCs express many different miRNAs already reported as being involved in neural regeneration—including miR-302/367 (Ghasemi-Kasman et al. 2018), miR-21 (Pan et al. 2021), miR-27a (Cai et al. 2016) and miR-152 (Das et al. 2010) (not shown) – our attention was specifically attracted by mir-514A-3p. Since mir-514A-3p has, thus far, not been considered much with respect to the nervous tissue, and was described as a molecule with potent involvement in controlling cell death/proliferation (Streicher et al. 2012; Jin et al. 2017; Quilang et al. 2022), it became the target of our analysis.

Interestingly, when comparing the levels of mir-514A-3p between normoxic and anoxic conditions, there is no noticeable difference in its levels. However, when faced with co-cultivated neural tissue, the expression of mir-514A-3p was significantly downregulated. This phenomenon was more pronounced in cells which were exposed to neurons for longer periods of time and it was equally present in normoxic and anoxic conditions (Fig. 5).

### Anoxia-Damaged Human Neurons can be Partly Rescued by hOMSCs and This Effect, Which Gradually Decreases Over Time, Depends on mir-514A-3p and SHP-2

To test if hOMSCs can rescue neurons damaged by anoxia, the following experiment was performed: human neurons (DIV50) were plated on the bottom of wells and then exposed to 6 h of anoxia. By using a transwell system which allowed us to add hOMSCs on top of the growing neurons, following groups of hOMSCs were added: (1) hOMSCs, (2) hOMSCs which were exposed to neural tissue for 28 days (hOMSC28), (3) hOMSCs with blocked expression of mir-514A-3p (hOMSC-mir), (4) hOMSCs with blocked expression of mir-514A-3p together with an inhibitor of SHP-2 (hOMSC-SHP-2) and (5) hOMSCs which were exposed to neural tissue for 28 days and which overexpressed mir-514A-3p (hOMSC28 +). Cell survival was measured in two time points: 6 h after co-cultivation of neurons and hOMSCs in anoxic condition (total length of anoxia 12 h) and following 12 h of reoxygenation.

The first significant finding was that hOMSCs which were added after 6 h of anoxia, and which were kept, together with neurons, in anoxic conditions for additional 6 h, improved the survival of neurons for 65% (Fig. 3A, Supplement Fig. 1B). However, when we added hOMSCs which were exposed to neural tissue for 28 days, these cells did not exhibit any rescuing capability (Fig. 3A, Supplement Fig. 1C). Another group of cells whose rescue capacity was tested were those in which we downregulated mir-514A-3p. Surprisingly, those cells could also not rescue anoxia-damaged neurons (Fig. 3A, Supplement Fig. 1D). However, PHPS1, a potent inhibitor of SHP-2, was added concurrently with these cells, their rescuing ability was re-established (Fig. 3A, Supplement Fig. 1E). Similarly, a significantly improved rescuing ability was achieved by hOMSCs exposed to neural tissue for 28 days, and which were modified to express mir-514A-3p (Fig. 3A, Supplement Fig. 1F). A very similar observation, yet with less pronounced significance of positive effects, was found after 12 days of reoxygenation. Therein, hOMSCs were much more potent in rescuing anoxia-damaged neurons than cells which were just exposed to neurons for 28 days. This capability was found to be mir-514A-3p and SHP-2-dependent (Fig. 6).

When LDH release was measured, as another parameter for quantifying cell damage and rescue, a significant improvement has been found in a group treated by hOMSCs and a group treated with hOMSCs which were, following 28 days of co-cultivation with neurons, transfected with miR-514A-3p (Fig. 7).

### hOMSCs Transplanted to Mice Affected by Stroke Accelerate and Improve Their Recovery

**Fig. 8:**
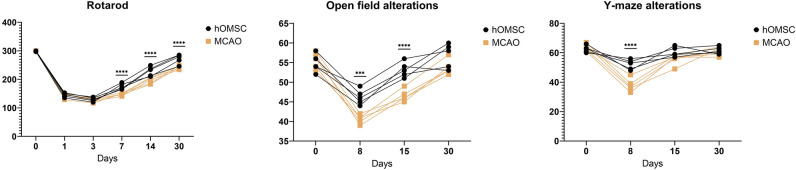
After inducing a stroke on Day 0, the mice received either 1 million of hOMSCs or just the cell medium on Day 2. The rotarod reveals a dramatic loss in motoric and coordination capability which was much better regained in the group which received cells on Days 7, 14 and 30. The difference between treated and placebo-treated group was as well visible in the Open field test, 8 and 15 days after the onset of stroke. This difference was not present 30 days after stroke. Y-maze test revealed significant differences between the groups 8 days after stroke, while 15 and 30 days after stroke, the cell and placebo-treated group did not differ

Since in vitro experiments revealed that hOMSCs are capable of surviving conditions with decreased levels of oxygen—coupled with the fact that they secrete growth factors and miRNAs which are, at least partly, responsible for rescuing in vitro anoxia-damaged human neurons—we performed a pilot experiment on mice affected by stroke. Out of 20 mice which underwent MCAO, we have selected 10 with rather uniform signs of impairment (minimum 10 out of 18 points for motoric and sensoric impairment). Two days after the onset of stroke, 1 million of hOMSCs were transplanted into five mice, near the cortex affected by ischemia. All results are visible in Fig. 8. The rotarod test revealed that both 1 and 3 days after stroke induction, which corresponds to 1 day before and 1 day after cell transplantation, all animals exhibited measurable motoric and coordination deficits. 7 days after the onset of stroke, i.e. 5 days after cell transplantation, a significant difference in score was observed in animals which received cell transplantations, as compared to those which were received just the culturing medium. The same difference was also mirrored 14 and 30 days after the onset of stroke. With that in mind, it is also important to notice that animals treated by cells reached an average recovery of 93% on Day 30. This was not the case for the control group, which, on average, recovered up to 82% of the condition before the stroke.

The open field test revealed significant improvements in cell-treated animals 8 and 15 days after the onset of stroke. Interestingly, no significant differences between treated and untreated animals in this test was observed 30 days after the stroke. Similar results have been obtained with the Y-maze test. While the treated animals scored much better than the untreated ones 8 days after the onset of stroke, there was no significant difference on Days 15 and 30.

### Improved Recovery of Mice Affected by Stroke After Transplantation of hOMSC is not Strongly Linked to Reduction of Apoptosis

**Fig. 9:**
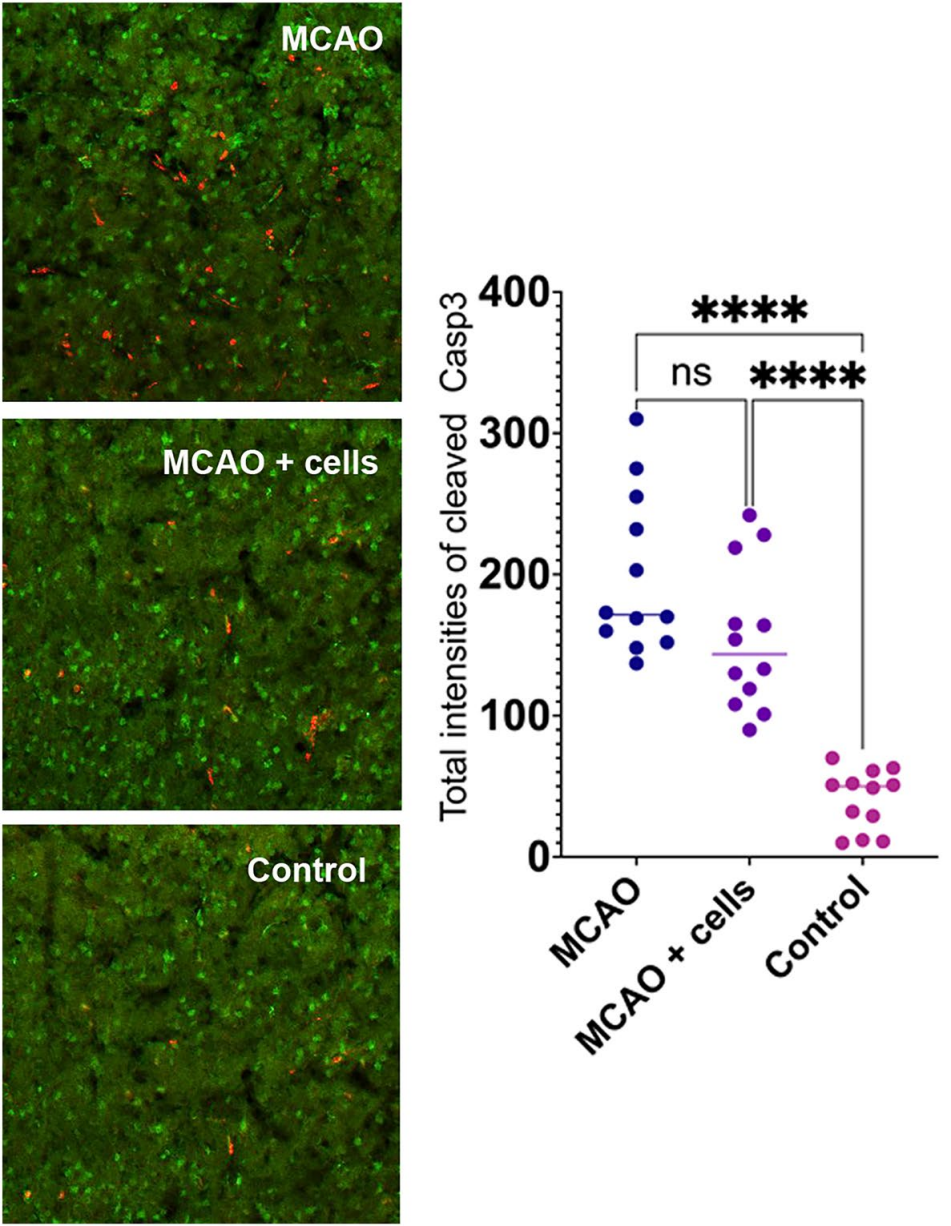
Immunohistochemical analyses of peri-infarct region of the mouse brain affected by stroke revealed that animals which received hOMSC seem to have lower total intensity of the cleaved Caspase 3, but this difference was not statistically significant

To analyse if improved recovery of mice after transplantation of hOMSC is linked to decreased apoptosis, serial sections of mice brain followed by immunocytochemistry against cleaved Casp3 was performed. Although the total intensity of cleaved Caspase 3 was smaller in animals which received hOMSC, this difference was not statistically significant (Fig. 9).

## Discussion

Following initial enthusiasm, stem cell therapy faced some serious disappointments in many clinical trials. However, a careful comparison of cases with varied degrees of success revealed that a precise selection of the source of cells, their standardization and defining of precise criteria for patient inclusion yielded more significant results (Mitrečić and al. 2009; Bohl et al. 2016; Parmar and al. 2019; Mitrečić et al. 2020, 2022; Rascón-Ramírez et al. 2021; Ejma et al. 2022).

And indeed, several recently published results of advanced-stage clinical trials reported positive effects of stem cells in patients affected by stroke (Jaillard et al. 2020; Muir et al. 2020; Lee et al. 2022). These beneficial effects of stem cells or their products mostly included improved cell survival and reduced inflammation (Shen et al. 2010; Mitrečić 2011; Mitrečić et al. 2012; Mine et al. 2013; Hribljan et al. 2019; Andjus et al. 2020).

With all that being said, it became clear that the source of stem cells remains one of the factors with the highest influence on the success of a clinical trial. As such, the goal of our work was to perform in vitro and in vivo tests on hOMSCs in order to facilitate detailed information on the possible suitability of these cells for treatment of ischemic brain disease. Since their discovery, hOMSCs were successfully tested in several pathological conditions and, among others, they showed positive effects in mitigating spinal cord damage and the symptoms in a Parkinsonian rat model (Ganz et al. 2014a, b; Lopez-Letayf et al. 2022). Accordingly, this is the first study which analysed their healing potential in pathological conditions linked to a lack of oxygen in neural tissue. Apart from hypothesized positive effects, we aimed to get insight into the molecular pathways in the background of their action. Although it has been already partially reported that hOMSCs secrete some growth factors (Ganz et al. 2014a, b), our study was designed starting from a specific question whether their profile would be influenced by a niche organized by neural tissue. Such type of data lacks in the literature or exists only for limited time points (Nicaise et al. 2011; Petschnik et al. 2011; Kim et al. 2014).

For this purpose, we exposed hOMSCs to human neurons, obtained from IPSCs, using a transwell system. Interestingly, we found that neural cells strongly influence hOMSCs by promoting their differentiation while downregulating expression of genes specific for pluripotency and growth factors. Although it is expected that transplanted stem cells which survive and differentiate lose at least part of their regenerative potential, this is, to the best of our knowledge, the first in vitro proof of scale of such transformations.

Our next question was linked to capability of hOMSCs to face decreased levels of oxygen. Starting from the fact that neuronal differentiation, including cells originating from the oral cavity might prefer hypoxic conditions, we hypothesized that hOMSCs might exhibit high resistance to lack of oxygen (Scully et al. 2016; Gugliandolo et al. 2019). And indeed, when compared to hIPSCs, hOMSCs appeared to be more robust when faced with anoxia. This confirmed our expectations that they might represent a good choice for cell-based therapy of brain hypoxia. Since, in parallel with analyses of their RNA profile, we performed a screening of miRNAs secreted by hOMSCs, we also found that some of the detected miRNA are typical for cells of early neuronal progenitors, like miR-302/367, miR-21, miR-492, miR-27a, miR-152, and miR-595. Since miR-514a-3p was reported as a strong regulator of proliferation and apoptosis, it was the main focus of our study (Streicher et al. 2012; Jin et al. 2017; Quilang et al. 2022). Moreover, it has also been reported that miR-514a-3p is involved in promotion of apoptosis through the nuclear factor kappa B pathway (Özata et al. 2017). At the same time, miR-514a-3p acts on SHP-2, ubiquitously expressed cytoplasmic tyrosine phosphatase, which is involved in proliferation and differentiation (Qu 2000; Lee et al. 2008; Batth et al. 2018). Most interestingly, it was recently shown that overexpression of miR-514a-3p blocks SHP-2 (Quilang et al. 2022). Additionally, studies demonstrated that overexpression of SHP-2 reduces neuronal differentiation and neurogenesis, alongside promoting apoptosis (Chitranshi et al. 2017).

In other words, it seems that miR-514a-3p in cells can, at some specific moments, act as a pro-apoptotic factor which is, for example, downregulated in tumours. Contrastingly, in cells in which pro-apoptotic signal over SHP-2 has been activated, miR-514a-3p inhibits cell death. And indeed, our experiments demonstrated that effect. When neurons damaged by 6 h of anoxia were co-cultured by hOMSCs for the next 6 h, a positive effect on their survival was observed only if miR-514a-3p was present. However, when we blocked SHP-2 by PHPS1, cell survival in anoxia significantly increased, despite the lack of miR-514a-3p. Importantly, that effect was not achieved by only performing the blocking of SHP-2, without adding hOMSCs. This suggests that miR-514a-3p is a factor which improves cell survival over inhibiting SHP-2 in the case of anoxia. At the same time, when SHP-2 is pharmacologically blocked, hOMSCs improve survival of neurons over some still unknown mechanisms. This phenomenon is present only in hOSMCs which were not influenced by neurons, i.e. those in which neurons did not cause the downregulation of miR-514a-3p.

Improvement of the health condition of experimental animals after ischemic or inflammatory damage of the CNS has been reported with many types of cells and is not limited to human neural stem cells (Mine et al. 2013), rat neural stem cells (Mitrečić et al. 2010) and adipose tissue mesenchymal cells (Bakreen et al. 2021). To test if accelerated and/or improved recovery can be induced by hOSMCs, we transplanted 1 million of hOMSCs to animals in which stroke was induced by MCAO. In congruence with what was observed in vitro, hOMSCs did, indeed, accelerate and improve the recovery. Performed Rotarod test revealed that animals’ motoric condition and coordination capabilities are regained faster following hOMSC transplantation. This effect seems to last up to Day 30, which was the last point in which we noticed a difference. Generally, hOMSC-treated animals recovered better than untreated ones. Interestingly, and in contrast to the results obtained with the Rotarod test, open field and Y-maze test results exhibited a different pattern. While on Day 8, where motoric recovery plays a dominant role, cell-treated animals performed better in both open field and Y-maze, no significant difference was observed for the final time point. Although we have shown that hOMSC-treated animals do regain higher levels of motoric capabilities, it is important to recognize that the results from the open field and Y-maze tests could have been influenced by the ratio between fear and curiosity and on memory. It seems that these two features are successfully regained during the normal course of recovery and no improvement in their values was observed following hOMSCs transplantation.

In conclusion, our results implicate hOMSCs as a potent candidate for clinical trials on patients affected by stroke. Although only a part of their beneficial action was discovered and described herein, the clear and measurable benefits our study has demonstrated bring new optimism into the field of regenerative medicine.

## Supplementary Information

Below is the link to the electronic supplementary material.

Supplementary file1 (TIF 17471 kb)—Immunocytochemical visualization of human neurons obtained from IPSC (DIV60) which were exposed to 6 hours of anoxia, followed by 6 hours of growth with various types of hOMSCs (Map2-red). In congruence with quantification of cell survival (Fig. 6) and LDH release (Fig. 7), here it is seen that samples treated by hOMSCs (B) and by hOMSCs which were either supported by a pharmacological block of SHP-2 (E) or which, following 28 days of co-culturing with neurons, were modified to overexpress miR-514-3p (F) exhibited improved neuronal morphology compared to the samples in which hOMSCs were previously co-cultured with neurons for 28 days (C) and in those in which miR-514-3p was inhibited (D). Scale bar: 100 µm.

## Data Availability

The datasets generated during the current study are available in the Dataverse repository of the Harvard University and can be assessed here: https://dataverse.harvard.edu/privateurl.xhtml?token=d454a384-5157-4136-9dba-d0e9fc33bfd7.
