# Supplementary figures and images for: Human Oral Mucosa Stem Cells Increase Survival of Neurons Affected by In Vitro Anoxia and Improve Recovery of Mice Affected by Stroke Through Time-limited Secretion of miR-514A-3p
Source: Cell Mol Neurobiol. 2022 Sep 9;43(5):1975–88. doi: 10.1007/s10571-022-01276-7 (PMC10287825; doi:10.1007/s10571-022-01276-7)

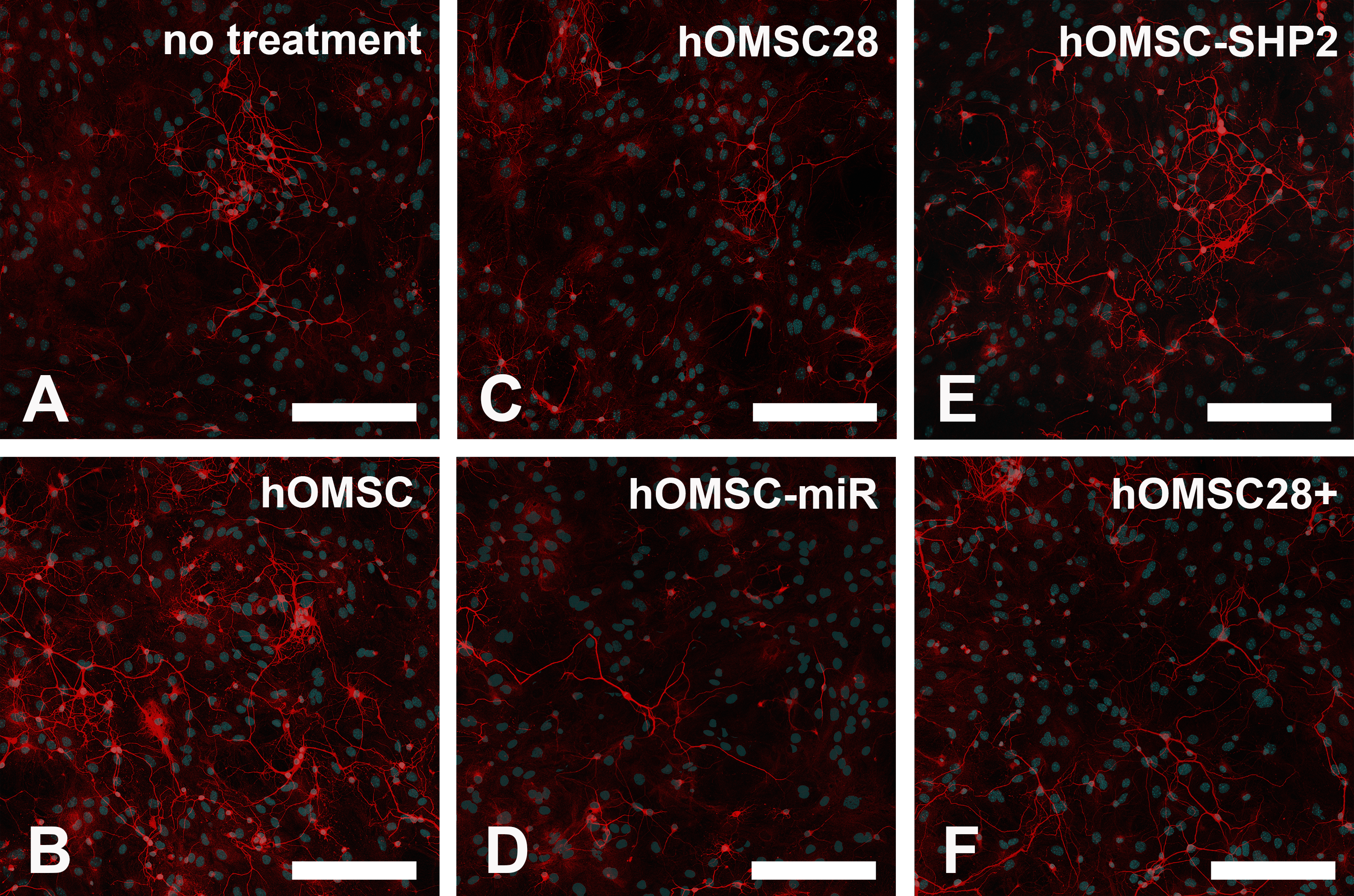

Supplement: Supplementary file 1 — Supplementary file1 (TIF 17471 kb)—Immunocytochemical visualization of human neurons obtained from IPSC (DIV60) which were exposed to 6 hours of anoxia, followed by 6 hours of growth with various types of hOMSCs (Map2-red). In congruence with quantification of cell survival (Fig. 6) and LDH release (Fig. 7), here it is seen that samples treated by hOMSCs (B) and by hOMSCs which were either supported by a pharmacological block of SHP-2 (E) or which, following 28 days of co-culturing with neurons, were modified to overexpress miR-514-3p (F) exhibited improved neuronal morphology compared to the samples in which hOMSCs were previously co-cultured with neurons for 28 days (C) and in those in which miR-514-3p was inhibited (D). Scale bar: 100 µm. [file 10571_2022_1276_MOESM1_ESM.tif]
